# Characterization of HIF‐1α/Glycolysis Hyperactive Cell Population via Small‐Molecule‐Based Imaging of Mitochondrial Transporter Activity

**DOI:** 10.1002/advs.201700392

**Published:** 2018-01-09

**Authors:** Yang Wang, Xingyun Liao, Jianguo Sun, Bin Yi, Shenglin Luo, Tao Liu, Xu Tan, Dengqun Liu, Zelin Chen, Xin Wang, Chunmeng Shi

**Affiliations:** ^1^ Institute of Combined Injury State Key Laboratory of Trauma, Burns and Combined Injury Chongqing Engineering Research Center for Nanomedicine College of Preventive Medicine Third Military Medical University Chongqing 400038 China; ^2^ Cancer Institute of PLA Xinqiao Hospital Third Military Medical University Chongqing 400037 China; ^3^ Department of Anesthesia Southwest Hospital Third Military Medical University Chongqing 400038 China

**Keywords:** cancer stem‐like cells, drug carriers, HIF‐1α/glycolysis, mitochondrial transporters, small molecules

## Abstract

The characterization of cancer stem‐like cells (CSCs) has profound implications for elucidating cancer biology and developing treatment strategies. Although surface markers are already used to identify CSCs, the expression of these markers is controversially linked to the phenotypes in different types of tumors and does not represent all functionally relevant of CSCs. Very recently, hyperactive HIF‐1α/glycolysis metabolic pathway is recognized as a master regulator of CSCs. In this study, a near‐infrared fluorescent small‐molecule, IR‐780, is identified for the exclusive characterization of human CSCs through the HIF‐1α/glycolysis dependent mitochondrial transporter ABCB10's activity. The results identified for the first time that ABCB10 is involved in the preferential uptake of IR‐780 in CSCs, which is regulated by HIF‐1α via the direct interaction with the binding site of ABCB10 gene promoter region. In addition, IR‐780 is demonstrated to conjugate with anticancer drug 5‐fluorouracil to act as a potential drug delivery carrier for CSC‐targeted therapy. Thus, the studies provide a new rational approach independent of surface markers to characterize CSCs via small‐molecule‐based imaging of HIF‐1α/glycolysis hyperactive metabolic pathway dependent mitochondrial transporter's activity, which holds promise for the further development of CSCs targeted diagnostic and therapeutic strategies.

## 1 Introduction

Cancer is continuously to be the deadly disease worldwide and recent researches hypothesize that a small subset of cancer stem‐like cells (CSCs), behave like stem‐cells with self‐renewal capacity, are giving rise to heterogeneous hierarchical organization of cancer cells and responsible for disease recurrence and therapeutic resistance.^1^, ^2^ Thus, the characterization of the CSC population is critical to develop more specific therapeutic strategies for cancer treatment.^3^ Currently, the routine methods using displayed surface makers are widely applied to target and characterize CSCs in different types of tumors.^4^ However, the expressions of surface markers are controversial in different types of tumors, which may even cause conflicting results in different of researches.^5^ Further, the most commonly used isolation methods based on surface markers do not represent a general functionally relevant of the CSC population.^6^ Thus, alternative strategies based on CSC functional properties would have profound implications for the development of future CSCs‐targeted therapeutic strategies.

Aerobic glycolysis is the basic metabolic characteristic of cancer cells, and has profound effects on cancer cell proliferation, biosynthesis and the initial stages of tumorigenesis.^7^ Recently, hyperactive glycolysis was found to be the preferred metabolic program in cancer cells and was regarded as the fundamental factors leading to the promotion of CSC self‐renewal and undifferentiation.^8^ Moreover, HIF‐1α was recognized to be the central driver that set off the cascade of events in cancer cell metabolic switch.^9^ The HIF‐1α regulatory pathways are also demonstrated to directly modulate stem cell functions and have been recognized as a master regulator of CSCs.^10^ Thus, targeting the functional activities of glycolytic metabolism and HIF‐1α relevant regulating pathways in CSC is supposed to represent a promising strategy to characterize CSCs.[[qv: 8a,11]] However, there are lack of reliable methods or agents for targeting CSCs by specific metabolism features and regulating pathways. In this study, we have identified a mitochondrial‐targeting small‐molecule fluorophore, IR‐780, to characterize CSCs via imaging the mitochondrial transporter activity which was regulated by hyperactive HIF‐1α/glycolysis regulating pathway. Furthermore, we also covalently conjugated IR‐780 with 5‐fluorouracil (5‐FU) to investigate the potential of IR‐780 as a carrier to deliver chemical drugs specific to CSCs. Thus, our data offer a novel approach independent of surface markers to characterize the CSC populations based on the HIF‐1α/glycolysis metabolic pathway. We also verify IR‐780 as a potential carrier to deliver antitumor drugs for the development of CSCs‐targeted therapeutic strategies.

## 2 Results and Discussion

### 2.1 IR‐780 Characterizes the Cancer Stem‐Like Cell Population

**Figure 1:**
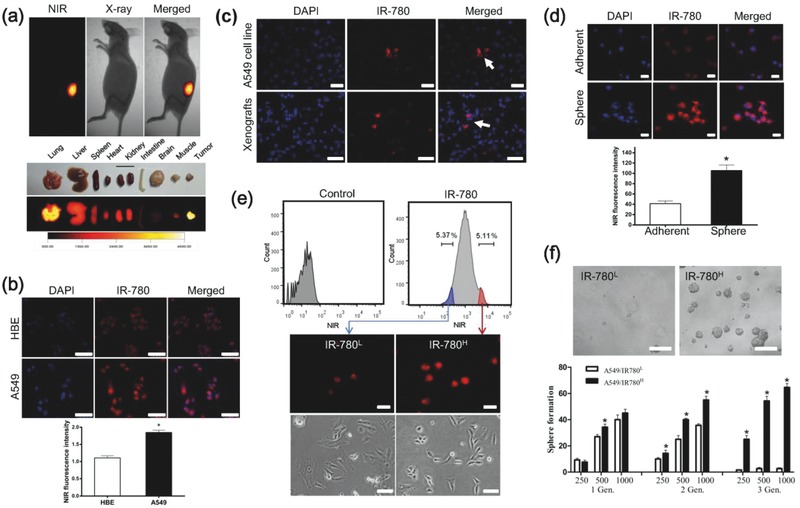
Characterization of cancer stem‐like cells by IR‐780. a) Preferential accumulation of IR‐780 in the mouse xenografts pre‐established with A549 cells, and the fluorescence imaging of dissected organs. The animal was subjected to imaging with the Kodak in vivo FX Pro imaging system. b) Analysis of NIR fluorescence intensity in normal cells and cancer cells after incubation with IR‐780, *n* = 5; scale bars: 100 µm. c) Localization of IR‐780‐enhanced cells in the cultured cancer cells (top) and xenograft tissue (bottom). Nuclei were stained with DAPI; scale bars: 100 µm. d) IR‐780 preferred to accumulate in the spheres cultured cells when compared with adherent cultured cells. *n* = 5; scale bars: 50 µm. e) Cancer cells were incubated with IR‐780 (2.5 × 10^−6^ m, 15 min) and subsequently sorted into two populations IR‐780^H^ cells (red) and IR‐780^L^ cells (blue) based on NIR fluorescence intensity by flow cytometry (BD FACSAria II), and the images of sorted cancer cells (below); scale bars: 50 µm. f) Representative images of spheroids formed in serum free medium, and statistical sphere numbers, *n* = 5; scale bars: 250 µm .* *p* < 0.05.

As the well‐known energy metabolism organelles, mitochondria determine the aberrant energetic metabolism and cell fate regulation in cancer cells^12^ and play fundamental roles in the initiation and progression of cancer.^13^ Therefore, the cancer‐specific alterations of phenotypic and functional properties in mitochondria have emerged as intriguing targets for tumor targeting and treatment.^14^, ^15^ In this study, we have identified a mitochondrial‐targeted small‐molecule fluorophore, IR‐780, which displayed CSC‐targeting property in human cancers. IR‐780 is a near‐infrared (NIR) fluorescent small‐molecule with emission at the NIR region that specifically targets cancer cells in vivo and in vitro (**Figure** **1**a,b). Furthermore, we observed that there was a small fraction of cells (≈5%) showed with significantly enhanced fluorescence intensity in different types of cancer cells (Figure 1c; Figure S1a,b, Supporting Information). Meanwhile, the fluorescence of IR‐780 was found to be more evident in spheres cultured cancer cells as compared with that in adherent cultured cells (Figure 1d). Because spheres culture is a traditional CSCs enrichment method, the results suggest a relationship between IR‐780 and cancer stem‐like cell population. To further assess stem‐like cell characteristics in IR‐780‐enriched cancer cells, A549 human lung cancer cells were incubated with 2.5 × 10^−6^ m IR‐780 for 15 min and sorted by a flow cytometry (BD FACSAria II) with 650 nm excitation and 780 nm emission. We set the analytical threshold to a 5% fluorescent signal for IR‐780 to distinguish the IR‐780‐enriched cancer cells according to previous data, and the sorted 5% high fluorescent cells were named IR‐780^H^ cells, while the 5% low fluorescent cells were called IR‐780^L^ cells. The fluorescence of these two cancer cell populations were confirmed under the NIR fluorescence microscope (Figure 1e). Then, the spheres‐formation assay was performed to determine the self‐renewal ability of cancer stem‐like cells. IR‐780^H^ cancer cells displayed significantly higher spheres‐forming efficiency than their counterpart IR‐780^L^ cells and expanded more efficiently in subsequent serial propagations (Figure 1f). Thus, these results demonstrated a rational new method to characterize CSCs using the NIR fluorophore IR‐780 independent of conventional surface markers.

### 2.2 IR‐780^H^ Cells Display Features of CSCs In Vitro

**Figure 2:**
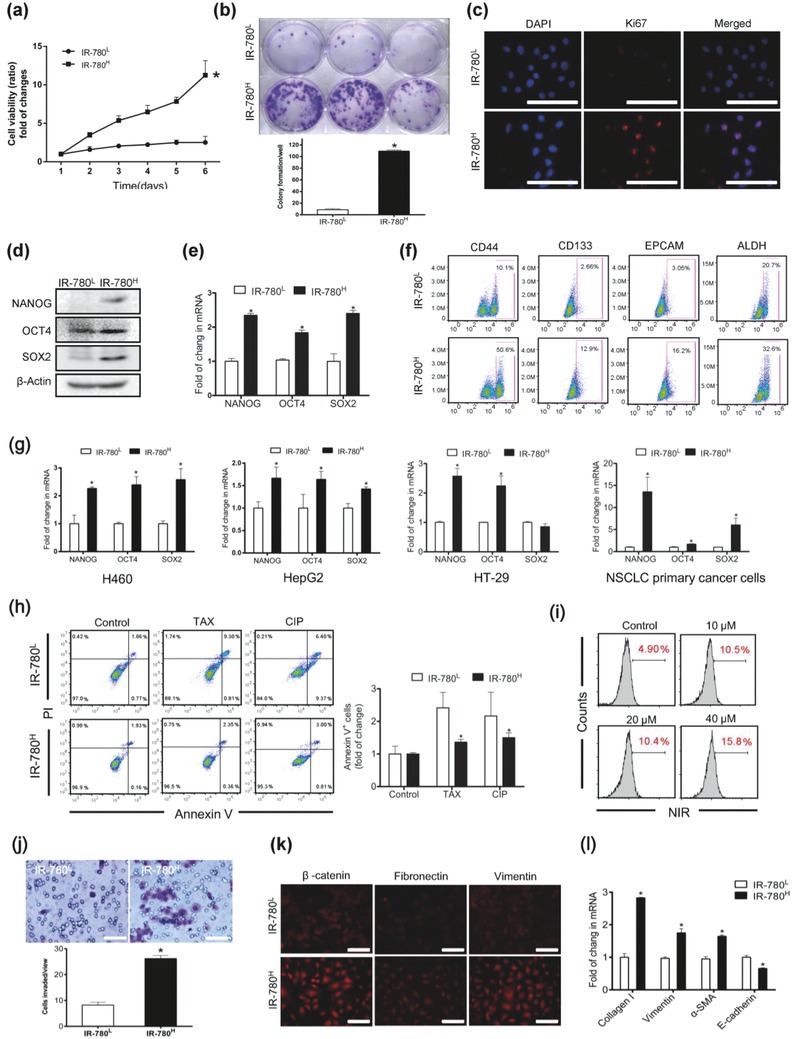
In vitro identification of cancer stem‐like cell features. a) The sorted cancer cells proliferation was detected in the complete medium. *n* = 6. b) Images of colony formation efficiency. *n* = 3. c) Immunofluorescence staining for Ki67 (red). Nuclei were stained by DAPI; scale bars: 10 µm. d) Western‐blot analysis of pluripotency‐associated genes, NANOG, OCT4, and SOX2 expression. e) Real‐time qPCR analysis of pluripotency‐associated genes expression. Data shown are normalized for GAPDH. *n* = 3. f) Flow cytometry analysis for the expressions of CSC markers in IR‐780^L^ and IR‐780^H^ cancer cells. g) Real‐time qPCR analysis expression of pluripotency‐associated genes in H460, HepG2, HT‐29 cancer cells, and isolated primary NSCLC cells. *n* = 3. h) Analysis of Annexin V staining in sorted cancer cells after treated with TAX and CIP. *n* = 3. i) The frequency of IR‐780^H^ cells was significantly increasing after cell were treated with increasing concentration of CIP. j) Representative images and statistical analysis of the migrating cells. *n* = 5; scale bars: 200 µm. k) Immunofluorescence staining for EMT relative markers in IR‐780^L^ and IR‐780^H^ cancer cells; scale bars: 200 µm. l) Real‐time qPCR analysis of EMT markers expression in the sorted cancer cells. *n* = 3. * *p* < 0.05.

To further confirm the stem‐like cell properties of IR‐780^H^ cancer cells, different phenotypical and functional features of CSCs were tested. The IR‐780^H^ cancer cells displayed superior proliferative potential (**Figure** **2**a), formed more colonies (Figure 2b) in complete culture medium and contained more proliferating Ki67^+^ cells (Figure 2c). Real‐time quantitative PCR (qPCR) analysis and Western blotting revealed that IR‐780^H^ cancer cells expressed a higher level of the pluripotency‐associated genes NANOG, OCT4, and SOX2 (Figure 2d,e). The common CSC surface markers CD44, CD133, EPCAM, and ALDH were variably upregulated in IR‐780^H^ cancer cells (Figure 2f). IR‐780^H^ cells sorted from different types of cancer cell lines and non‐small‐cell lung carcinoma (NSCLC) primary cancer cells (isolated from clinical specimen) were also proven to overexpress pluripotency‐associated genes (Figure 2g). All these results confirmed the stem‐like cell characteristics in IR‐780^H^ cancer cells subpopulation. IR‐780^H^ cells were also demonstrated to be more tolerant to the chemotherapy drugs Taxol (TAX) and cisplatin (CIP) (Figure 2h). As previous studies have reported the enrichment of cancer stem‐like cells after chemotherapy,^16^ our data also indicated the relative number of IR‐780^H^ cells in cancer cells were increased after CIP treatment (Figure 2i). In addition, the transwell assay revealed the enhanced migration capacity of IR‐780^H^ cancer cells (Figure 2j), and these metastatic IR‐780^H^ cancer cells expressed high levels of epithelial‐to‐mesenchymal transition (EMT) markers (Figure 2k,l). In conclusion, the sorted IR‐780^H^ cancer cells from cancer cell lines and primary human cancer cells displayed the phenotypical and functional features of CSCs, including enhanced proliferative potential, expression of pluripotency‐associated genes, resistance to chemotherapy, and high migration capacity. All of these features were also consistently confirmed in another lung cancer cell line H460 (Figure S2, Supporting Information).

### 2.3 IR‐780^H^ Cells Possess High Tumorigenicity In Vivo

**Figure 3:**
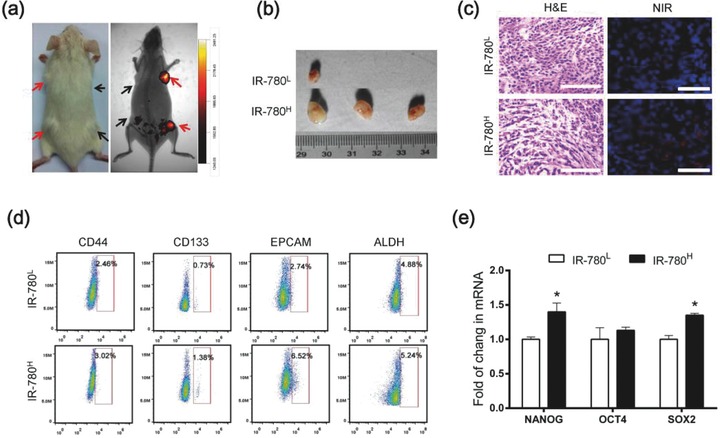
In vivo tumorigenicity of IR‐780^H^ cancer cells. a) NIR fluorescent image of tumor formation in NOD/SCID mice after s.c. injected with the same number of IR‐780^H^ cells (red arrows) and IR‐780^L^ cancer cells (black arrows). b) Representative photographs of dissected tumors from NOD/SCID mice (*n* = 3). c) H&E staining and fluorescent imaging of frozen section in dissected tumor tissues from NOD/SCID mice; scale bars: 100 µm. d) Flow cytometry analysis of indicated CSC markers expressions in the IR‐780^L^ and IR‐780^H^ cancer cells from NOD/SCID mice xenografts. e) Real‐time qPCR analysis for the expression of pluripotency‐associated genes in cancer cells isolated from NOD/SCID mice xenografts, *n* = 3. * *p* < 0.05.

Cancer stem‐like cells are defined as a subset of cancer cells that retain extensive self‐renewal potential and have the ability to recreate the heterogeneity of the original tumor.^17^ To confirm whether IR‐780^H^ cancer cells possess tumor initiating ability, human lung cancer cells A549 were labeled with IR‐780 and sorted using flow cytometry as previous described. An in vivo tumorigenicity assay was performed by subcutaneously injecting the same number of sorted IR‐780^H^ and IR‐780^L^ cancer cells into nonobese diabetic/severe combined immunodeficient (NOD/SCID) mice. **Figure** **3**a revealed that IR‐780^H^ cells formed obvious tumor xenografts, which were also verified by the in vivo whole‐body NIR fluorescent imaging after tail vein injection of IR‐780. The IR‐780^H^ cancer cells initiated tumor formation in almost all transplanted mice and only 10^3^ cells could form tumors within one month, whereas IR‐780^L^ cells formed limited tumors (Figure 3b and **Table** **1**). Histopathological analysis indicated that tumor morphology was slightly different and the IR‐780^H^ tumor frozen section displayed more obvious NIR fluorescent cancer cells (Figure 3c). Intriguingly, the common CSC surface markers (CD44, CD133, EPCAM, and ALDH) were upregulated in cancer cells isolated from IR‐780^H^ tumor tissues (Figure 3d), and the pluripotency‐associated genes (NANOG, OCT4, and SOX2) were also slightly upregulated as compared with cancer cells from IR‐780^L^ tumors (Figure 3e). Taken together, the results indicated that the sorted IR‐780^H^ cancer cells display functional features of CSCs and have the ability to recreate the heterogeneity of the original tumor. These data further confirm that the NIR fluorescent small‐molecule IR‐780 could be used to characterize cancer stem‐like cell population independent of surface markers.

**Table 1:** The in vivo tumorigenicity of subcutaneously injected IR‐780^L^ and IR‐780^H^ cancer cells. (Tumors take after one month (# tumors/# injections.))

No. of cells injected		10^5^	10^4^	10^3^	Freq.	*P* value
A549	IR‐780^L^	2/3	3/6	1/3	<0.0001%	0.00038
	IR‐780^H^	3/3	5/6	3/3	0.011%	

### 2.4 IR‐780 Uptake is Depend on HIF‐1α/Glycolysis Metabolism

**Figure 4:**
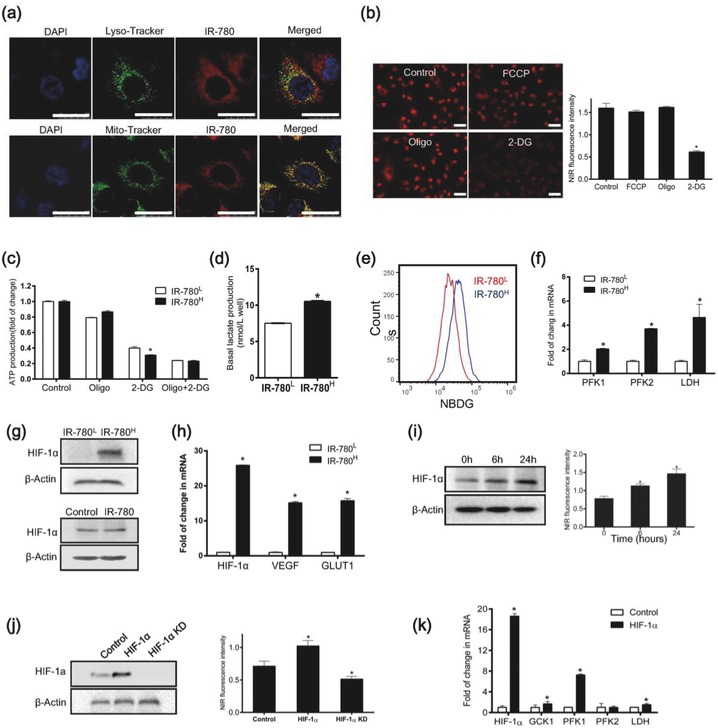
Mechanistic study of the preferential accumulation of IR‐780 in CSCs. a) Confocal images for the localization of IR‐780 (red) relative to the indicated organelles markers (green, Lyso‐tracker and Mito‐tracker); scale bars: 25 µm. b) Analysis of IR‐780 uptake in cancer cells treated with 2.0 × 10^−6^ m FCCP, 2.0 × 10^−6^ m Oligo and 250 × 10^−3^ m 2‐DG, respectively, *n* = 5; scale bars: 50 µm. c) ATP production was detected after treated with FCCP, Oligo or both of compounds, *n* = 5. d) Basal lactate production was tested, *n* = 3. e) Cellular glucose uptake by 2‐NBDG incorporation was detected by flow cytometry. f) Real‐time qPCR analysis for glycolysis relative genes, *n* = 3. g) Western‐blot detection of HIF‐1α expression in sorted cancer cells (top) and A549 cells treated with IR‐780 for 15 min (bottom). h) Real‐time qPCR analysis of HIF‐1α and its target genes expression, *n* = 3. i) HIF‐1α expression was examined after treatment with CoCl_2_ (left), and the determination of IR‐780 intakes (right), *n* = 5; scale bars: 50 µm. j) HIF‐1α expression in cancer cells transfected with HIF‐1α plasmid or siRNA, respectively (left), and the uptake of IR‐780 in these cells (right), *n* = 5; scale bars: 50 µm. k) Real‐time qPCR analysis for glycolytic genes in cancer cells transfected with HIF‐1α plasmid, *n* = 3. * *p* < 0.05.

As the sorted IR‐780^H^ subpopulation of cancer cells were confirmed to display all CSC features, mechanistic assessment of the preferential accumulation of IR‐780 in cancer cells might provide new insights into CSC biology. With the help of its intrinsic NIR fluorescence, IR‐780 was shown to accumulate in the mitochondria of cancer cells by colocalization with the mitochondrial specific fluorescent probe Mito‐tracker Green (**Figure** **4**a). Thus, mitochondrial mass in the sorted cancer cell was measured to investigate whether mitochondrial mass was the cause of enhanced NIR fluorescent intensity in IR‐780^H^ cells. As shown in Figure S3 of the Supporting Information, there was no difference in mitochondrial mass between the two cancer cell populations. To further investigate the factors affecting cellular uptake of IR‐780, cancer cells were treated with mitochondrial transmembrane potential depolarizer FCCP, glycolysis inhibitor 2‐deoxy‐d‐glucose (2‐DG) or oxidative phosphorylation inhibitor oligomycin (Oligo), respectively. As shown in Figure 4b, glycolysis inhibitor 2‐DG significantly decreased the cellular uptake of IR‐780, while FCCP and Oligo did not, suggesting that glycolysis may play an important role in the IR‐780 uptake. The cellular metabolic patterns of IR‐780^H^ and IR‐780^L^ cancer cells were further investigated. The cellular ATP levels were detected after treatment with Oligo, 2‐DG, and both compounds. Figure 4c showed that the inhibition of glycolysis by 2‐DG led to a marked decrease of ATP production in IR‐780^H^ cancer cells, demonstrating a stronger dependence on glycolysis in IR‐780^H^ cells. In addition, lactate productions and glucose uptakes were increased in IR‐780^H^ cancer cells (Figure 4d,e), and the key glycolytic enzymes PFK1, PFK2, and LDH were also upregulated in IR‐780^H^ cancer cells (Figure 4f). These data indicated that enhanced glycolysis in IR‐780^H^ cancer cells might be the key factor mediating the preferential uptake of IR‐780 in CSCs. Further studies indicated that the HIF‐1α protein expression was upregulated in IR‐780^H^ cancer cells, and the expression was not affected by the IR‐780 pretreatment (Figure 4g). Real‐time qPCR analysis also confirmed that HIF‐1α and its downstream target genes Glut1 and VEGF were upregulated (Figure 4h). Cobalt chloride (CoCl_2_), a chemical hypoxia mimicker, was used to increase the cellular HIF‐1α level. As shown in Figure 4i, both HIF‐1α level and IR‐780 uptake were increased in a time‐dependent manner with the treatment of CoCl_2_. Moreover, Figure 4j showed that the overexpression of HIF‐1α via plasmid transfection increased the uptake of IR‐780, while knockdown of HIF‐1α via short interfering RNA (siRNA) transfection reduced IR‐780‐uptake. These data indicated that cellular HIF‐1α activity played an important role in the cellular uptake of IR‐780. Figure 4k confirmed that the expression of glycolysis‐related genes was correlated with the overexpression of HIF‐1α via plasmid transfection. Thus, our results revealed that the HIF‐1α mediated enhanced glycolysis metabolic pathway played important role in the uptake of IR‐780 in CSCs.

### 2.5 IR‐780 Uptake is Mediated by Mitochondrial Transporter ABCB10

**Figure 5:**
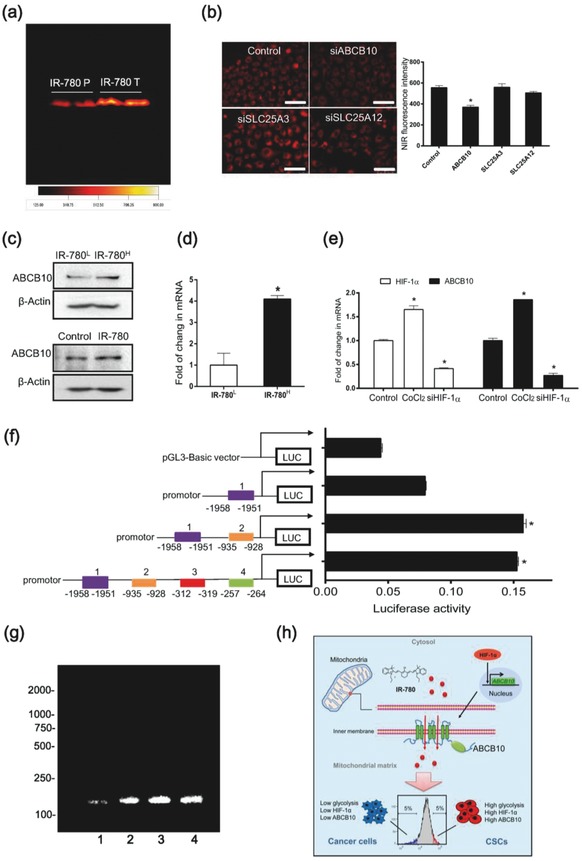
Mitochondrial transporter ABCB10 mediated the uptake of IR‐780 in CSCs. a) NIR fluorescence imaging for mitochondrial protein separated by SDS‐PAGE (IR‐780 T: tumor, IR‐780 P: para‐carcinoma). b) Images of cancer cells pretreated with siRNA specifically targeted to ABCB10, SLC25A3 and SLC25A12 (left) and fluorescent intensity was quantified by Leica LAS AF Lite software, *n* = 5; scale bars: 100 µm. c) Western‐blot analysis of ABCB10 expression in sorted cancer cells (top) and in A549 cancer cells treated with IR‐780 for 15 min (bottom). d) Real‐time qPCR analysis of ABCB10 expression in sorted cancer cells, *n* = 3. e) Real‐time qPCR analysis of HIF‐1α and ABCB10 expression in cancer cells treated with CoCl_2_ (200 nm, 24 h) or siRNA specific to HIF‐1α, *n* = 3. f) Promoter activities of a series of constructs determined by luciferase assay. Firefly luciferase activity was normalized to the corresponding Renoilla luciferase activity values, *n* = 3. g) ChIP assay was performed using chromatin from A549 cancer cells treated with CoCl_2_ for 24 h. PCR product was observed in the anti‐ABCB10 (lane **4**) but not in the normal IgG ChIP (lane **1**), ABCB10 promoter specific DNA was observed in the Input (lane **3**), and lane **2** was the positive control (anti‐RNA polymerase II). h) Schematic diagram demonstrating the roles of HIF‐1α/glycolysis‐dependent mitochondrial transporter ABCB10 in the characterization of CSCs by IR‐780. * *p* < 0.05.

To further investigate the underlying molecular mechanisms of IR‐780 uptake by cancer cells, mitochondrial proteins were extracted from the tumor xenografts after intraperitoneal injection with IR‐780. Taking advantages of the intrinsic NIR fluorescent property, the potential binding proteins were separated by SDS‐PAGE electrophoresis (**Figure** **5**a), and further identified using the liquid chromatography tandem mass spectrometry. Three of mitochondrial inner membrane transporters ABCB10, SLC25A3, and SLC25A12 were identified as putative transporters. Among them, ABCB10 was verified to be the key factor affected the uptake of IR‐780 by siRNA transfections (Figure 5b). Western blotting assay (Figure 5c) and real‐time qPCR (Figure 5d) further demonstrated that the expression of ABCB10 in the IR‐780^H^ cancer cells was higher than it is in IR‐780^L^ cells. The ABCB10 is a member of the ATP binding cassette energy‐dependent transporters and is demonstrated to responsible for iron import to the mitochondria in previous report.^18^ Our study identified ABCB10 as an oncogenic relevant gene and was involved in the uptake of IR‐780 by cancer cells. Interestingly, we further observed that the overexpression of HIF‐1α by CoCl_2_ treatment was correlated with the increase of ABCB10 expression levels, while knockdown of HIF‐1α by siRNA reduced the expression of ABCB10 (Figure 5e), suggesting a relationship between HIF‐1α and ABCB10. To investigate whether HIF‐1α directly regulated the ABCB10 gene, we predicted the potential binding sites in the ABCB10 promoter region using bioinformatics analysis based on the JASPAR database, and 5 putative binding sites (**1**, **2**, **3**, **4**, and **5**) were predicted at the upstream of the transcription initiation site of the ABCB10 gene spanning from −1958 to −257 bp (Table S1, Supporting Information). Figure 5f showed that the luciferase activity in site **2** was the highest, indicating GCACGTGT was the critical binding site for transcription factor. ChIP analyses were performed to further confirm that HIF‐1α directly bind to GCACGTGT of ABCB10 gene promoter region (Figure 5g). These results provide direct evidences that the high level of HIF‐1α in CSCs instinctually upregulating mitochondrial transporter ABCB10's activity by facilitating the cis–trans interactions of the binding sites in the promoter region to mediate the cellular uptake of IR‐780 (Figure 5h).

### 2.6 IR‐780 as a Drug Delivery Carrier for CSCs Targeted Therapy

**Figure 6:**
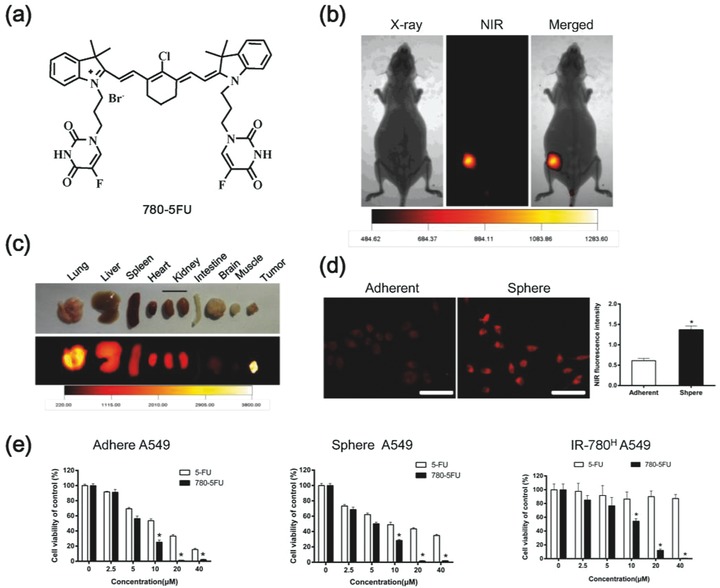
Synthesis of 780–5FU for CSC‐targeted therapy. a) Structure of 780–5FU containing the parent structure of IR‐780 and antitumor chemical moiety. b) Images of the preferential accumulation of 780–5FU in the nude mice xenografts pre‐established with A549 cancer cells. The animal was subjected to imaging with the Kodak in vivo FX Pro imaging system after systemic administration of 780–5FU (0.2 mg kg^−1^). c) The NIR fluorescent imaging of dissected organs further confirmed the preferential accumulation of 780–5FU in tumor. d) Analysis of NIR fluorescence intensity in adherent and spheres cultured A549 cancer cells after incubation with 780–5FU; *n* = 5, scale bars: 100 µm. e) Cell viability was detected after incubation with 5‐FU or 780–5FU in adherent, spheres cultured and IR‐780^H^ A549 cancer cells, respectively; *n* = 6. * *p* < 0.05.

Due to the intrinsic CSCs targeting property, we further investigated the potential of IR‐780 as a drug delivery carrier to develop CSCs‐targeted therapeutic strategies. 5‐FU is a critical systemic chemotherapy drug widely used in the treatment of cancer patients including colorectal cancer, gastric cancer, liver cancer, breast cancer, lung cancer, and skin cancer.^19^ However, the low specificity of 5‐FU to cancer cells leads to severe side effects. In the study, 5‐FU was chosen as the starting material to synthesize a new heptamethine cyanine dye (780‐5FU) containing the parent structure of IR‐780 and an antitumor chemical moiety (**Figure** **6**a). It was prepared according to the procedure delineated in Figure S5 of the Supporting Information. The structure of 780–5FU was confirmed by ^1^H NMR, ^13^C NMR, ^19^F NMR, and high resolution mass spectrometer (HRMS), as described in the Supporting Information. The in vivo NIR fluorescence imaging was performed after intravenous injection of 780–5FU by tail veins and indicated the tumor targeted property of 780–5FU in A549 human lung cancer nude mice model (Figure 6b) and dissected organs (Figure 6c). In addition, 780–5FU was also demonstrated to prefer to accumulate in the spheres cultured of A549 cancer cells when compared with adherent cancer cells (Figure 6d). The anticancer capability of 780–5FU was compared with 5‐FU in A549 cancer cell, spheres cultured A549 cancer cells and IR‐780^H^ cancer cells, respectively. As shown in Figure 6e, 780–5FU displayed better anticancer activity than 5‐FU not only in A549 cancer cells but also in spheres cultured A549 cancer cells and IR‐780^H^ cancer cells. Thus, these studies indicated that IR‐780 could be employed as a potential chemical drugs delivery carrier specifically to the cancer stem‐like cells, which would improve clinical treatment efficacy and reduce the side effects.

## 3 Conclusion

In summary, the identified NIR fluorescent small‐molecule, IR‐780, as a newly strategy for the characterization of CSCs has enormous advantages over the current CSCs‐targeted strategies. IR‐780 was demonstrated to preferentially uptake by the CSCs through the mitochondrial membrane transporter ABCB10, which was upregulated by HIF‐1α interacting with the binding sites of the ABCB10 gene promoter region. Thus, the NIR fluorescent imaging of mitochondrial transporter's activity by IR‐780 is actually reflecting the metabolic features and HIF‐1α regulating pathway for the characterization of CSCs. In addition, IR‐780 was demonstrated to conjugate with 5‐FU and exhibited enhanced CSCs‐targeting therapeutic effects. Although more in‐depth studies are still needed to determine the CSCs‐targeting mechanisms, our studies have provided a reasonable approach, independent of surface markers, to characterize by imaging of the functional transporters' activity, and provided a potential carrier for the delivery of anticancer drugs toward CSCs, which may hold significant promise for the further development of CSCs‐targeted therapeutic strategies.

## 4 Experimental Section

*Agents and Apparatus*: Mito‐tracker Green and Lyso‐tracker Green were purchased from Molecular Probe. 2‐deoxy‐d‐glucose (2‐DG), oligomycin, and carbonylcyanide p‐trifluoromethoxy phenyl‐hydrazone (FCCP) were purchased from Sigma‐Aldrich. All the other chemicals and solvents with analytical grade were obtained from Aladdin Chemistry Reagent Company. ^1^H NMR, ^13^C NMR, and ^19^F NMR spectra were recorded on a Bruker 400 MHz spectrometer. HRMS was performed in a Bruker BioTOFIIIQ.

*Cell lines and Clinical Samples*: The human lung cancer (A549 and H460), colorectal cancer (HT‐29), and liver cancer (HepG2) were purchased from the American Type Culture Collection and cultured in recommended medium with 10% FBS in the incubator at 37 °C with 5% CO_2_. Human NSCLC tissues were obtained from patients (Southwest Hospital, Third Military Medical University) who received surgical resection with written informed consent. The fresh tissues were collected and washed with PBS, minced, mechanically digested by gentle MACSTM Dissociator (Miltenyi Biotec) and collagenase IV (Worthington) with DNAase (Sigma) digested, cells were primary cultured in DMEM medium containing 15% FBS with 1% penicillin/streptomycin and expansion after removal of fibroblasts. Acquisition and use of these clinical samples were approved by the Ethics committee of the Third Military Medical University.

*Cellular NIR Fluorescence Detection*: Cells were incubated with 2.5 × 10^−6^ m IR‐780 diluted in basic DMEM medium at 37 °C for 15 min under dark condition. For the adherent cell, cells were washed with PBS and observed by confocal laser scanning fluorescence microscopy (Lecia) with 633 nm laser to excite IR‐780 with emission of 750–800 nm. The NIR fluorescent intensity was calculated by Leica LAS AF Lite software. For the suspension cell, cells were resuspended in PBS with 1 × 10^−3^ m EDTA and measured by the flow cytometry (BD FACSVerseTM, BD Biosciences) with 633 nm excitation and 780 nm emission.

*Spheres Formation Assay*: Spheres formation assay was performed by culturing the same numbers of isolated cells in the serum free DMEM/F12 medium with B‐27 supplement (gibico), 20 ng mL^−1^ of EGF (PEPRO TECH), 10 ng mL^−1^ bFGF (PEPRO TECH) for about two weeks, medium was replaced every 3 d. For serial propagation, the spheres were harvested and dissociated into single cells by trypsin (Hyclone) and then recultured in the serum free medium as previously described. The numbers of mammospheres in each well was calculated under microscopy.

*Transwell Assay*: For transwell assay, the 24‐well transwell plates (Corning) with transwell chambers contained the membrane with 8 µm pores were used after rehydrated for 2 h.^20^ 2 × 10^4^ cells in 100 µL serum free medium were seeded into the apical side of insert and 600 µL of DMEM plus with10% FBS was added into the basal side of the insert as the chemoattractant. After cultured for 8–12 h, cells on the apical side of the inserts were scraped off with sterile cotton. The basal side of the insert cells were fixed with 4% paraformaldehyde and stained with crytal violet solution. The numbers of migrated cells were counted from five different fields of inserts under microscopy.

*In Vivo Tumorigenicity Assay and NIR Imaging*: For in vivo tumorigenicity assay, the NOD/SCID mice (six weeks old, female, purchased from the laboratory animal center of the Third Military Medical University) were used. Animal protocols were in accordance with the “Animal Care and Committee Guidelines of the Third Military Medical University.” The IR‐780^H^ and IR‐780^L^ cancer cells were resuspended in DMEM medium with the same volume of Matrigel (BD Biosciences) and subcutaneously injected into the flank of each mouse. For in vivo NIR imaging, mice were tail vein injection with IR‐780 at a dose of 0.2 mg kg^−1^. After more than 24 h, mice were anesthetized by 1% pentobarbital sodium (Sigma) and the whole body NIR fluorescent imaging was taken using a Kodak In‐Vivo FX Profession Imaging System equipped with fluorescent filter sets (excitation/emission, 770/830 nm). Fluorescent images were coregistered with the anatomical X‐ray images in this system. All the settings were applied as described previously.[[qv: 15a]]

*RNA Interference*: siRNA targeting ABCB10 (siABCB10) SLC25A3 (siSLC25A3) SLC25A12 (siSLC25A12) and nonspecific siRNA were used to transiently knockdown these genes expression. The sequences of siRNA were as follows: ABCB10 siRNA: 5′‐CAGUGUGGCUGAGAUCCAATT‐3′, SLC25A3 siRNA: 5′‐GGCGCACAUCACUAUAUUUTT‐3′, SLC25A12 siRNA: 5′‐CCGAAAUUUAAGUCUCCUATT‐3′, and nonspecific siRNA: 5′‐UUCUCCGAACGUGUCACGUTT‐3′. The siRNA were transfection using lipofectamine 3000 (Invitrogen) according to the manufactures' instructions.

*Luciferase Reporter Activity Assay*: The putative HIF‐1α binding sites in ABCB10 promoter region were predicted according to the JASPAR database. For luciferase reporter activity assay, A549 cells were transfected with HIF‐1α plasmid and ABCB10 promoter constructs together with Renilla using lipofectamine 3000 (Invitrogen). Cells transiently transfected with the luciferase constructs were harvested at 48 h. The activities of firefly and Renilla luciferase in cell lysates were measured by a dual‐luciferase reporter assay system (Promega) using a varioskan LUX multifunctional reader (Thermo Fisher). The firefly luciferase to renilla luciferase ratios were determined and defined as the relative luciferase activity. All experiments were performed in triplicate.

*ChIP Assay*: The ChIP assay was performed to determine the association of HIF‐1α protein with ABCB10 promoter in A549 cancer cells treated with cobalt chloride (200 µm, 24 h). In brief, cells were sonicated on wet ice with Sonicator (50 W model equipped with a 2 mm tip and set to 30% of maximum power gave about 200–1000 bp length DNA fragments). Immunoprecipitation of crosslinked protein was performed with constant rotation at 4 °C overnight using HIF‐1α antibodies (Abcam). The eluates were pooled and heated at 45 °C for 2 h to reverse the formaldehyde crosslinks (4 µL 0.5 m EDTA, 8 µL 1 m Tris‐HCL and 1 µL Proteinase K). DNA fragments were purificated using spin columns. For standard end‐point PCR, 2 µL of the DNA sample and 32 cycles of amplification were used. 10 µL of each PCR reaction was analyzed by 4% agarose gel electrophoresis with a 2000 bp DNA size marker. The primer of ABCB10 was designed according to the result of Luciferase activity assay, ABCB10‐F: 5′‐GCACGATAAGGCGGGTGA‐3′, ABCB10‐R: 5′‐GCGCAAAGCCCGA GGAC‐3′, GAPDH‐F: 5′‐TACTAGCGGTTTTACGGGCG‐3′, GAPDH‐R: 5′‐TCGAACAGGAGGAGCAGAGAGCGA‐3′.

*Statistical Analysis*: All data were presented as the mean ± standard deviation. SPSS 13.0 statistical software was used to conduct all statistical analysis. One‐way analysis of variance was used to determine significance among groups. For statistical analysis of in vivo tumorigenicity, the limiting dilution analysis (LDA; http://bioinf.wehi.edu.au/software/elda/) was used according to the previous studies.^21^ A value of *p* < 0.05 was considered to be statistically significant.

## Conflict of Interest

The authors declare no conflict of interest.

## Supporting information

Supplementary
