# Supplementary material for: Characterization of HIF‐1α/Glycolysis Hyperactive Cell Population via Small‐Molecule‐Based Imaging of Mitochondrial Transporter Activity
Source: Adv Sci (Weinh). 2018 Jan 9;5(3):1700392. doi: 10.1002/advs.201700392 (PMC5867035; doi:10.1002/advs.201700392)
Supplement: Supplementary file 1 — Supplementary [file ADVS-5-1700392-s001.pdf]

Copyright WILEY-VCH Verlag GmbH & Co. KGaA, 69469 Weinheim, Germany, 2018.

ADVANCED SCIENCE
Open Access

Supporting Information

for *Adv. Sci.,* DOI: 10.1002/advs.201700392

Characterization of HIF-1α/Glycolysis Hyperactive Cell Population via Small-Molecule-Based Imaging of Mitochondrial Transporter Activity

*Yang Wang, Xingyun Liao, Jianguo Sun, Bin Yi, Shenglin Luo, Tao Liu, Xu Tan, Dengqun Liu, Zelin Chen, Xin Wang, and Chunmeng Shi**

Copyright WILEY-VCH Verlag GmbH & Co. KGaA, 69469 Weinheim, Germany, 2016.

Supporting Information

Characterization of HIF-1α/glycolysis Hyperactive Malignant Cell Population via Small-Molecule-Based Imaging of Mitochondrial Transporter Activity

*Yang Wang, Xingyun Liao, Jianguo Sun, Bin Yi, Shenglin Luo, Tao Liu, Xu Tan, Dengqun Liu, Zelin Chen, Xin Wang, and Chunmeng Shi**

Dr. Y. Wang, X. Liao, Dr. S. Luo, Dr. T. Liu, X. Tan, Dr. D. Liu, Dr. Z. Chen, Dr. X. Wang, Prof. C. Shi

Institute of Combined Injury, State Key Laboratory of Trauma, Burns and Combined Injury

Chongqing Engineering Research Center for Nanomedicine

College of Preventive Medicine

Third Military Medical University

Chongqing 400038, China

E-mail: shicm@sina.com

Prof. J. Sun

Cancer Institute of PLA, Xinqiao Hospital

Third Military Medical University

Chongqing 400037, China

Prof. B. Yi

Department of Anesthesia, Southwest Hospital

Third Military Medical University

Chongqing 400038, China

Synthesis of 780-5FU

5-Fluorouracil (1.560g, 12.0 mmol) was heated to dissolve in 1,1,1,3,3,3–hexamethyldisilazane (10.0 mL, 48.0 mmol) under argon. Then chlorotrimethylsilane (250 uL) was added and the solution was stirred for 4.5 h at 126°C. The reaction mixture was cooled and the excess solvent was removed under reduced pressure to yield yellow green oil. This oil immediately reacted with dried 1, 3-dibromoalkane (10 mL) under 105 °C for 3 h to obtain compound **1**(1.07 g, 35.5%).

Next, a reaction mixture containing 2,3,3-trimethyl-3H indole (583mg, 3.67mmol), **1** (460 mg, 1.83mmol) was reacted in 1,2-dichlorobenzene at 110°Cfor 10 h. After the reaction, the reaction mixture was washed with acetone andrecrystallizedin isopropyl alcohol to obtain the quaternary ammonium salt of indolium (**2**, 363 mg, yield 50%). Compound **3** (Bisaldehyde) was synthesized according to our previously reported method [1].

Finally, a reaction mixture containing **2** (290 mg, 0.71mmol) and bisaldehyde **3** (54 mg, 0.31 mmol) in a refluxed solution of toluene and n-butyl alcohol (10mL, 7:3) was allowed to react for 12 h. After the reaction, the solvent was removed under reduced pressure and the residue was purified by silica gel column chromatography to achieve synthesis of the target compound **4**, (186.6 mg, 30%). Its structure was confirmed by ^{1}H NMR, ^{13}C NMR, ^{19}F NMR, and HRMS. ^{1}H NMR (400 MHz, $CDCl_3$): δ 2.099(s, 12H,CH3), 2.266m(d, 2H, β-CH2), 3.089(m, 4H, CH2), 3.882(m, 4H, CH2), 2.484 (s, 4H, α-CH2), 2.738(s, 4H, CH2CH=), 4.120(t, 4H, N-CH2), 6.216(d, 2H, CH=CH), 7,169-7.413 (m, 8H, Ar-H), and 8.325(d, 2H, CH=CH). ^{13}C NMR(400 MHz, DMSO-d6): δ 174.223, 172.180, 171.925, 147.948, 147.930, 142.004, 141.021, 128.598, 126.156, 125.134, 122.478, 111.489, 101.573, 48.945, 43.629, 33.402, 27.434, 26.680, 25.788, 25.597, 24.123, 20.971, and 20.339. ^{19}F NMR(400 MHz, DMSO-d6): δ -169 ppm. HRMS (m/z): calc. 683.3616 $[M-Br]^+$, measured 683.3610$[M-Br]^+$.

Supplementary Data

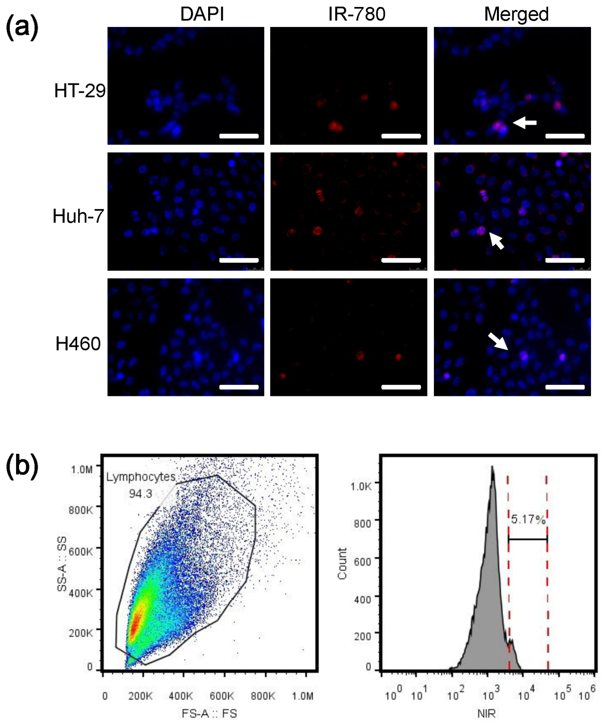

Figure S1. Identification of CSCs-targeting property in different types of cancer cells. a) Fluorescent images showing the localization of IR-780-enhanced cells in different types of cancer cells. Nuclei were stained with DAPI. Scale bars, 100 μm. b) Flow cytometry analyses the percentage of IR-780-enhanced cells in the heterogeneous cancer cells.

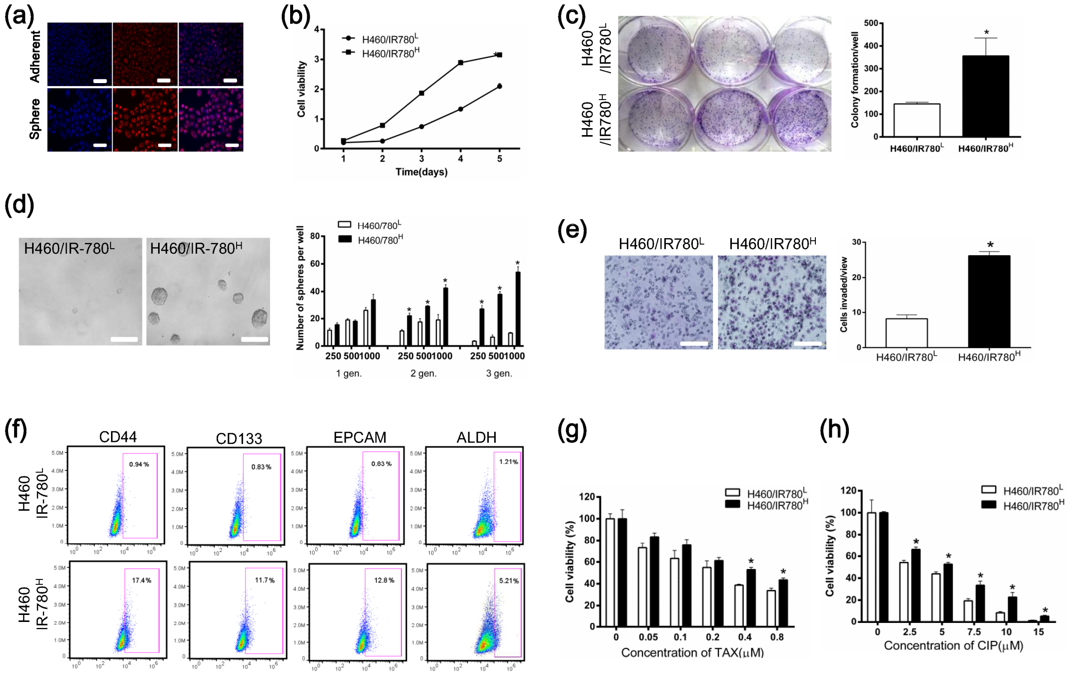

Figure S2. Identification of cancer stem-like cell properties in IR-780^{H} cells from H460 cell. a) IR-780 preferred to accumulate in the spheres cultured H460 cells. Scale bars: 50 µm. b) Cellular viability was tested in the complete medium (n = 6). c) Analysis of colony formation efficiency (n = 3). d) Analysis of spheroids formation in serum free medium over three generations (n = 5). Scale bars: 250 µm. e) Representative images of the cells migrating (n = 5). Scale bars: 250 µm. f) Real-time qPCR analysis of EMT relative markers in IR-780^{L} and IR-780^{H} cancer cells. Data shown are normalized for GAPDH expression (n = 3). g) Flow cytometry analysis of the indicated CSC markers expression in IR-780^{L} and IR-780^{H} cancer cells. h) The cell viability of sorted IR-780^{L} and IR-780^{H} cancer cells after treated with chemotherapeutic drugs TAX and CIP (n = 6, * $p < 0.05$).

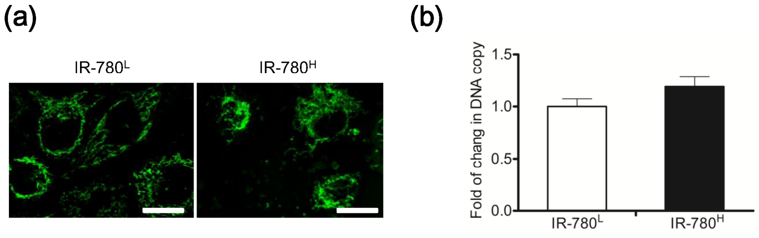

Figure S3. Detection of mitochondrial mass. a) Mitochondrial mass was compared by Mito-Tracker labeling in IR-780^H and IR-780^L cancer cells, scale bars: 25 μm. b) Real-time qPCR determination of mitochondrial DNA copy numbers in the cancer cells (n = 3).

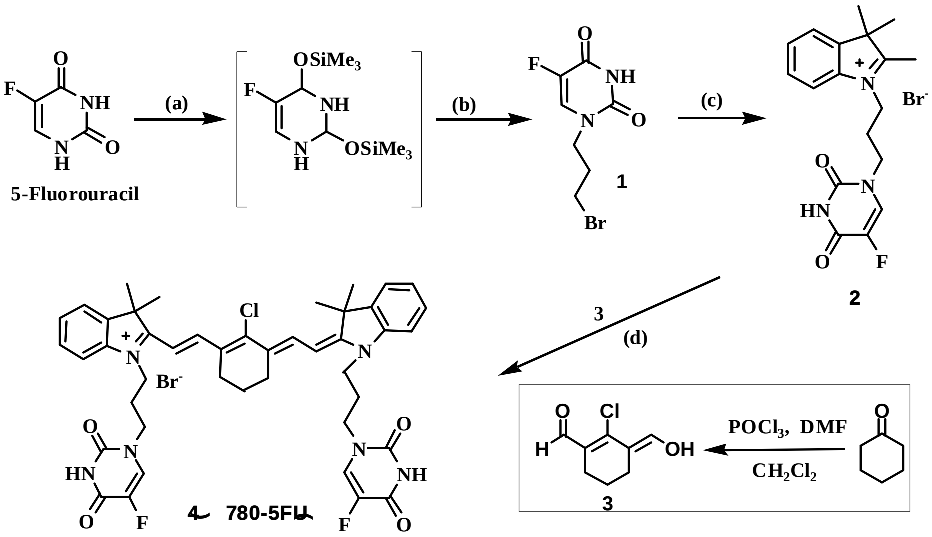

Figure S4. Synthetic route of 780-5FU. Reagents and conditions: (a)HMDS, TMSCl, 126 ℃, 4.5 h; (b) 1,3-dibromoalkane, 105 ℃, 3 h; (c) 2,3,3-trimethylindolenine, 1,2-dichlorobenzene, 110℃, 10 h; (d) bisaldehyde 3, n-butyl alcohol/toluene (7/3, v/v), reflux, 4 h.

Table S1. List of putative HIF-1α binding sites in ABCB10 promoter region, which were predicted according to the JASPAR database

Model name	Score	Relative score	Star	End	Strand	Predicted site sequence
HIF-1α	8.000	0.90455	257	264	-1	GAGCGTGC
HIF-1α	9.196	0.94024	312	319	-1	CGACGTGG
HIF-1α	10.184	0.96972	928	935	-1	ACACGTGC
HIF-1α	9.377	0.94564	928	935	1	GCACGTGT
HIF-1α	8.295	0.91337	1951	1958	-1	GGGCGTGG

Table S2. List of utilized primer sequence for real-time qPCR.

**Gene**	**Primer forward**	**Primer reverse**
NANOG	AGAACTCTCCAACATCCTGAACCT	TGCCACCTCTTAGATTTCATTCTCT
OCT4	CTTGCTGCAGAAGTGGGTGGAGGAA	CTGCAGTGTGGGTTTCGGGCA
SOX2	AGAACCCCAAGATGCACAAC	CGGGGCCGGTATTTATAATC
E-cadherin	TCCTCCCAATACATCTCCCTTCA	TCTCCGCCTCCTTCTTCATCATA
Vimentin	TTCGCCAACTACATCGACAAGG	TTCAAGGTCAAGACGTGCCAG
Collagen□	GATGTGCCACTCTGACTGG	ACATCGATGATGGGCAGGC
α-SMA	AGGGGGTGATGGTGGGAATG	GCCCATCAGGCAACTCGTAAC
PFK1	CACTGTGAGGATTGGCCTTAT	GTCACAGGTTGTGCAGATAGT
PFK2	TACAGTTGTGGCCTCCAATATC	GCATCTCCTCTGCACTCTTATC
GCK1	CCCACACCAAACTGCCTGTATTAG	TTGGTCAGTGTAGGTCGAACTCATG
LDH	CTTCCTGAGCCTTCCATGTATC	GATTATGTGAGCCCAAATTCAC
HIF-1α	GACAGCCTCACCAAACAGAG	CTCAAAGCGACAGATAACACG
VEGF	GCTACTGCCATCCAATCGAG	CTCTCCTATGTGCTGGCCTT
GLUT1	GTCACCATCCTGGAGCTGTT	GAAGGCCGTGTTGACGATAC
ABCB10	ACTCTCTTCCTTCCTAATGTATGCTTTCTG	CACCCAGTCCTTTCATCAGCT
SCL25A3	GCCGTGGAAGAGCAGTATAGC	GGGGGTCCACCTGCATTCTG
SLC24A12	CATGGAGCTTGTTCGTAA	AAGTTCTGCCAGGTTGTA
mtDNA	CAAACCTACGCCAAAATCCA	GAAATGAATGAGCCTACAGA
GAPDH	GGCATCCTGGGCTACACT	CCACCACCCTGTTGCTGT

Reference

[1] S. Luo, X. Tan, Q. Qi, Q. Guo, X. Ran, L. Zhang, E. Zhang, Y. Liang, L. Weng, H. Zheng, T. Cheng, Y. Su, C. Shi, *Biomaterials* **2013**, *34*, 2244.
